# Development of a High-Sensitivity Wireless Accelerometer for Structural Health Monitoring

**DOI:** 10.3390/s18010262

**Published:** 2018-01-17

**Authors:** Li Zhu, Yuguang Fu, Raymond Chow, Billie F. Spencer, Jong Woong Park, Kirill Mechitov

**Affiliations:** 1 School of Civil Engineering, Beijing Jiaotong University, Beijing 100044, China; zhuli@bjtu.edu.cn; 2 Department of Civil and Environmental Engineering, University of Illinois at Urbana-Champaign, Urbana, IL 61801, USA; yfu15@illinois.edu; 3 Epson Electronics America, San Jose, CA 95112, USA; rchow@eea.epson.com; 4 Anne M. and Nathan M. Endowed Chair in Civil Engineering, University of Illinois at Urbana-Champaign, Urbana, IL 61801, USA; 5 School of Civil and Environmental Engineering, Urban Design and Studies, Chung-Ang University, Seoul 06974, Korea; jongwoong@cau.ac.kr; 6 Department of Computer Science, University of Illinois at Urbana-Champaign, Urbana, IL 61801, USA; mechitov@illinois.edu

**Keywords:** high-sensitivity accelerometer, structural health monitoring, wireless smart sensor

## Abstract

Structural health monitoring (SHM) is playing an increasingly important role in ensuring the safety of structures. A shift of SHM research away from traditional wired methods toward the use of wireless smart sensors (WSS) has been motivated by the attractive features of wireless smart sensor networks (WSSN). The progress achieved in Micro Electro-Mechanical System (MEMS) technologies and wireless data transmission, has extended the effectiveness and range of applicability of WSSNs. One of the most common sensors employed in SHM strategies is the accelerometer; however, most accelerometers in WSS nodes have inadequate resolution for measurement of the typical accelerations found in many SHM applications. In this study, a high-resolution and low-noise tri-axial digital MEMS accelerometer is incorporated in a next-generation WSS platform, the Xnode. In addition to meeting the acceleration sensing demands of large-scale civil infrastructure applications, this new WSS node provides powerful hardware and a robust software framework to enable edge computing that can deliver actionable information. Hardware and software integration challenges are presented, and the associate resolutions are discussed. The performance of the wireless accelerometer is demonstrated experimentally through comparison with high-sensitivity wired accelerometers. This new high-sensitivity wireless accelerometer will extend the use of WSSN to a broader class of SHM applications.

## 1. Introduction

Structural health monitoring, combining various sensing technologies with data acquisition and processing capability, plays a significant role in assessing the condition of structures. The ability to continuously monitor the integrity of structures in real-time can provide increased safety for the public, particularly for the aging structures in widespread use today. Moreover, the ability to detect damage at an early stage can reduce the costs and down-time associated with repair of critical damage.

The shift of SHM research away from traditional wired methods toward the use of wireless smart sensor networks has been motivated by the many attractive features of wireless smart sensors. State-of-the-art WSSs offer the promise of wireless communication, onboard computation, low cost, less invasive installation, small size, and performance equivalent to that of their macro-scale counterparts [1,2,3]. These features enable the deployment of a dense array of sensors on structures, which can provide real-time information about the performance of civil infrastructure.

**Figure 1:**
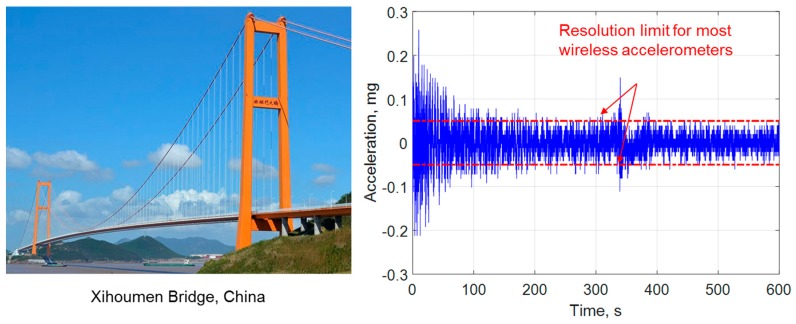
Lateral acceleration response at the midspan of the deck on the Xihoumen Bridge in China [5].

One of the most important structural responses employed by SHM systems is acceleration, and as a result, nearly all wireless smart sensors include accelerometers. Because WSSs traditionally rely on battery power, low-power MEMS-based accelerometers are typically employed. Many of these accelerometers were initially developed for automobile airbag and mobile phone applications, and as a result, have relatively low resolution compared to wired accelerometers used in SHM applications [4]. The demands of monitoring of civil infrastructure are severe. For example, Figure 1 shows the lateral acceleration of the deck at the midspan of the Xihoumen Bridge in China. Most wireless sensors are unable to measure these low-level structural vibration responses, undermining efforts to achieve successful SHM with WSSs.

Table 1 shows a summary of MEMS-based accelerometers employed for WSS that have been reported in the literature since 2006. A detailed description and comparison can be found in [6]. The resolution of the wireless MEMS-based accelerometers employed in Table 1 is primarily found to be larger than 0.05 mg for a 20 Hz bandwidth [7]. An exception is found in two of the most recently developed wireless nodes [8,9,10,11] which employ the SiFlex accelerometers manufactured by Colibrys (Yverdon-les-Bains, Switzerland). More specifically, Kohler et al. [8], employs three Colibyrs SiFlex 1500 single-axis accelerometers, which can achieve resolution of 1.4 μg and wide frequency range of 0.1–90 Hz for low-amplitude and low-frequency vibration measurement. Sabato et al. [10,11] developed a wireless node that employs a SiFlex 1600 single axis accelerometer, which is designed for micro-vibrations. This node uses Voltage to Frequency Conversion (V/F) instead of the Analog to Digital Conversion (ADC) to maximize the accelerometer’s performance. However, validation of the performance of these wireless accelerometers nodes in the low-frequency, ultra-low amplitude range has not been reported. Moreover, the SiFlex accelerometers employed in these two sensor systems have three problems: (i) a bipolar power supply of ±6V to ±15V is required, which is difficult to provide with standard DC power sources (i.e., batteries); (ii) the power draw is relatively high; and (iii) this sensor line is no longer commercially available.

This paper presents the development of a next-generation wireless accelerometer node for structural health monitoring applications. The M-A351, a high-resolution (0.06 μg/LSB) and low-noise (0.5 μg/$\sqrt{\mathrm{Hz}}$) tri-axial MEMS accelerometer manufactured by Seiko Epson Corporation (San Jose, CA, USA), is integrated with the Xnode, a powerful hardware platform with a robust software framework, to enable both campaign-style and long-term SHM. The M-A351 requires a unipolar 3.3 V power supply, which is ideal for use in battery-powered wireless sensor applications. Issues with both software and hardware implementation are discussed. This high-sensitivity wireless accelerometer enables collection of high fidelity acceleration data, specifically focusing on low-level accelerations. To demonstrate the efficacy of the device, its performance is compared experimentally with high-sensitivity wired piezoelectric accelerometers.

## 2. Xnode

This research leverages a next-generation wireless smart sensor platform, the Xnode; a brief overview of the Xnode in terms of hardware and software is provided in this section.

### 2.1. Hardware

**Figure 2:**
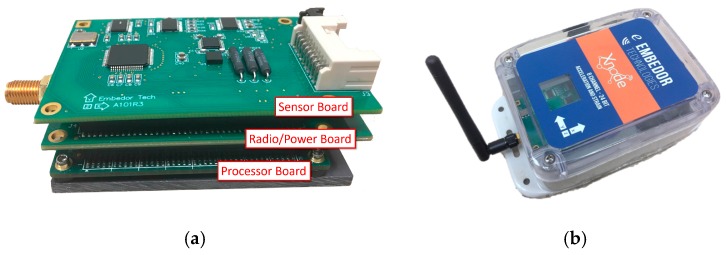
Xnode Smart Sensor: (**a**) 3-board stack; (**b**) Enclosure.

The standard Xnode consists of three printed circuit boards (PCB) which are: (i) the processor board; (ii) the radio/power board; and (iii) the sensor board [22], as shown in Figure 2. The processor board, a customized Mini4357 developed by Embest Technology (Shenzhen, China), features an LPC4357 microprocessor from NXP (Eindhoven, The Netherlands) that operates with a dual Cortex M4/M0 core (ARM, Cambridge, UK) at frequencies up to 204 MHz. This board can be used to execute on-board computation once data is acquired. The Mini4357 has 32 MB of SDRAM, which can be used for temporary data storage and processing. The Mini4357 has numerous general purpose input/output (GPIO) pins and peripheral interfaces such as serial peripheral interface (SPI) and inter-integrated circuit (I2C).

To extend the data storage size for high sampling rate applications, a microSD card can be plugged into the radio/power board, providing up to 4 GB of extensible storage. The radio/processor board includes a power management circuit that controls charging from wall power and solar panels. The radio transceiver in the radio/power board is a 2.4 GHz Zigbee radio for low-power wireless communication (AT86RF233, Atmel, San Jose, CA, USA). A radio booster has been added which allows communication to reach over 2 km line-of-sight with maximum transfer rates of 1 Mbps.

The standard sensor board employs a 24-bit ADC (ADS131E8, Texas Instruments (Dallas, TX, USA), which has eight channels of input that accommodates sampling rates up to 16 kHz. The sensor board incorporates a tri-axial analog accelerometer (LIS344ALH), leaving up to 5 channels available for external analog sensors.

The developed hardware is stacked together to comprise an integrated sensor node. The sensor node is packaged in an IP67 environmentally hardened enclosure (see Figure 2). Through the connectors on either side of the enclosure, the solar panel, wall-power, and external sensors can be easily attached.

### 2.2. Software

**Figure 3:**
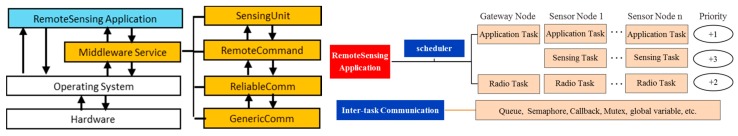
RemoteSensing application structure and its implementation in FreeRTOS.

The Xnode retains much of the successful SOA-based middleware functionality of the Illinois SHM Services Toolsuite developed for the Imote2 [25]. RemoteSensing, a fundamental distributed data acquisition application in the Toolsuite, is implemented using the FreeRTOS operating system [26]. Specifically, this application is used to help the coordination between gateway node and multiple sensor nodes to realize the remote sensing application. Sensor data is collected through RemoteSensing, which provides a basis for most applications in the ISHMP Toolsuite. RemoteSensing is supported by several middleware services [26], including SensingUnit, RemoteCommand, ReliableComm and GenericComm, as shown in Figure 3. 

A total of three types of tasks are defined for implementation of RemoteSensing in FreeRTOS, assigned with different priorities, as shown in Figure 3. Specifically, the *Sensing Task* has the highest priority to avoid missing data in the process of data acquisition. The *Radio Task* has the second highest priority to ensure the efficient communication between a gateway node and multiple sensor nodes. Meanwhile, several mechanisms are available to enable inter-task communication, including queues, semaphores, callback functions, mutexs, etc. Using FreeRTOS, different functionalities in RemoteSensing are isolated within separate tasks, and these tasks are executed efficiently, by means of a preemptive, priority-based scheduler.

The Xnode platform uses standard C language for application development, in place of a custom nesC variant on the Imote2, which allows for much simpler portability of code libraries and applications to and from the Xnode platform. Many platform-independent libraries and numerical algorithms implemented in C language can be used directly, without modification, with the Xnode. Additionally, the radio communication is based on the IPv6 protocol stack, widely adopted by the Internet of Things (IoT) community, opening the door to integration of heterogeneous devices inside a single WSSN.

## 3. High-Sensitivity Accelerometer Integration

To enable reliable measurement of accelerations found in many civil infrastructural applications, a high-sensitivity accelerometer is integrated with the Xnode platform. This section describes the challenges encountered in this process, along with the associated resolutions.

### 3.1. Design Challenges

Strong structural excitations, such as earthquakes and hurricanes, can result in high levels of structural response which are readily captured by general-purpose wireless accelerometers, because the signal-to-noise ratio (SNR) of the structural responses is large. However, most structural responses that can be measured during routine monitoring are low-level ambient vibration responses. Ambient vibration is generated due to a variety of external sources such as nearby traffic, normal wind-loading, machinery inside the structures, etc. While ambient vibration data can be useful for vibration-based SHM, usually the vibration level is too small to be captured with WSS nodes (see Figure 1 and Table 1). New wireless accelerometers that have µg resolution are needed for WSS technology to reach its full potential.

Vibration of civil infrastructure is usually dominated by the first few modes. However, damage often precipitates itself in the higher modes, whose amplitude is much smaller. Thus, a high dynamic range is needed so that the important higher mode responses can be measured. Additionally, considering the limited power available to WSS nodes, the accelerometer should be able to be run on a low-voltage and low-current power source and should be able to be powered off when not in use. The interaction between ground and digital grounds in the PCB design also must be considered to ensure that the WSS doesn’t introduce unwanted noise into the accelerometer measurements.

Therefore, the three typical design challenges should be addressed to the development of the wireless accelerometer on the Xnode: (1) identifying an appropriate accelerometer of high resolution, low noise output and low power consumption; (2) designing a stable and low-noise PCB for physical integration of the accelerometer with the Xnode; and (3) developing effective and precise driver for the programmable accelerometer to achieve its functionality on the Xnode.

### 3.2. M-A351 Specifications

**Figure 4:**
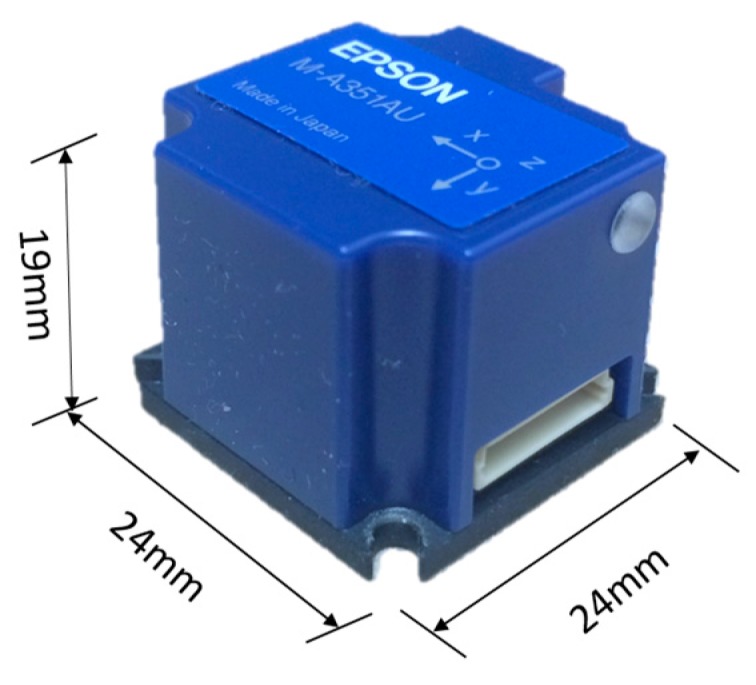
M-A351 accelerometer.

The M-A351 manufactured by Epson, shown in Figure 4 [27], is a high-sensitivity digital accelerometer that has the potential to address the first challenge of finding the appropriate MEMS accelerometer. More specifically, the M-A351 has superior performance in the aspects of noise level, resolution, dynamic range, data acquisition (sampling rate) and power requirements. It enables measurement bandwidths of up to 100 Hz, and a data output rate of up to 1000sps. Moreover, the measurement resolution and the noise level is only 0.06 μg/LSB and 0.5 μg/$\sqrt{\mathrm{Hz}}$ (average), respectively, and the output mode of each acceleration axis can be selected from acceleration, tilt angle, and tilt angle speed. The accelerometer is a highly accurate and stable tri-axial accelerometer equipped with detection elements made from quartz crystal micro-fabrication technology and can operate over a wide temperature range due to a built-in temperature compensation. Because the M-A351 is a digital sensor, no additional ADC is required, and interaction between analog and digital ground doesn’t need to be considered in the PCB design. 

The device has an internal FIR filter that uses a Kaiser Window with selectable filter Taps (128, 256 or 512) and programmable cutoff frequencies (Fc = 5, 10, 20, 50 or 100 Hz). Use of the M-A351 with serial digital SPI (M-A351AS) and UART (M-A351AU) output simplifies the design of high performance measurement systems, while reducing the overall system cost, weight, and power consumption. The main features of the M-A351 are given in Table 2.

### 3.3. PCB Design

**Figure 5:**
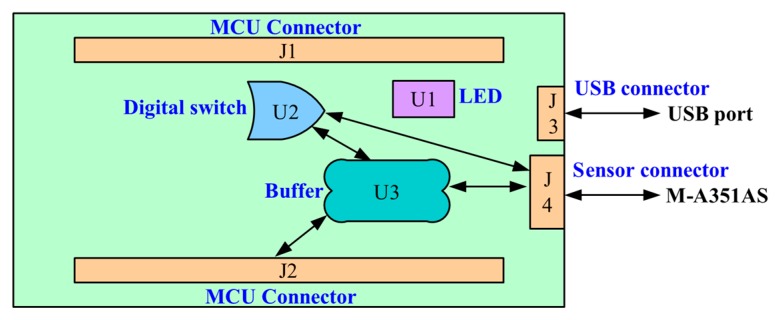
Design concept of high-sensitivity sensor board.

To address the challenge of physical integration of the M-A351 accelerometer and the Xnode, a printed circuit board, designated SHM-H2, was designed. The three key functions of the SHM-H2 are to communicate control messages to the M-A351, retrieve sensor measurement data, and control the on-board power-down circuitry to the sensor. Figure 5 illustrates the design concept of the SHM-H2, where J and U represent connectors and components, respectively. A total of four connectors are employed, including two MCU connectors, a USB connector, and an M-A351AS connector (the accelerometer with serial digital SPI output is selected in this study). The two MCU connectors (J1 and J2) are used for the connection among the processor board, the radio/power board and the SHM-H2 board. The USB connector (J3) provides the physical interface for the USB port, which can supply the power for the SHM-H2 board and provide a command interface to the MCU. The M-A351AS accelerometer, which is not directly PCB-mounted, can be connected with the SHM-H2 board through a wired connection (J4). Three electronic components, an LED SMLP34RGB (U1), a Complementary Metal–Oxide–Semiconductor (CMOS) single-pole and single-throw switch ADG821 (U2), and a buffer SN74LVC244A (U3) are implemented.

Designing partial power-down of electronic subsystems or gating power to board sub-circuits can be a non-trivial task, with care needed to prevent leakage currents and handle proper power sequencing. Unless specifically designed internally with special-purpose I/O circuitry, a typical standard CMOS devices such as the M-A351 requires external logic and power up/down sequencing to avoid unintentional current draw during power-down and malfunction after power-up. Therefore, the fundamental task of this partial power-down and power sequencing function is to keep I/O interface pins of the M-A351A device at GND (0V) level when it is powered off. This approach prevents a leakage current path from the external powered MCU signals to the device’s I/O pins through the internal ESD protection diodes to internal VDD power rail of the IC devices (which will be at GND level when powered down).

To address the design challenge of powering down the accelerometer when not in use, a CMOS switch was used to control the power supply to the M-A351AS through a GPIO. When the voltage of the GPIO is high, the switch is connected to power the buffer (U3) and the M-A351AS. The buffer (U3) is located between the MCU signal pins and the M-A351AS to block the current when the voltage of the power pin is low. The buffer is a generic off-the-shelf CMOS IC, but is specifically chosen because it features partial power-down circuitry and current blocking when the VCC power pin is at GND. The buffer is able to block the potential leakage current path and safely maintain the voltage of the MCU signal pins when the accelerometer is powered off. The LED is used to provide an indication of the Xnode’s current state via a combination of three colors, red, green, and blue.

**Figure 6:**
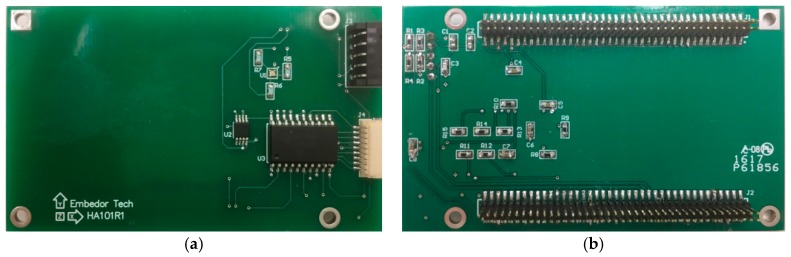
PCB design of high-sensitivity sensor board. (**a**) Top side; (**b**) Bottom side.

Figure 6 shows the realized PCB for the high-sensitivity accelerometer. The accelerometer connectors, USB connectors, and other components are soldered on the top side, while the MCU connectors, companion resistors and capacitors are soldered on the bottom side. Because the accelerometer is a digital device, only a digital ground has been included in the PCB design.

### 3.4. Driver of M-A351AS

In addition to development of the sensor board hardware, an effective and precise driver is needed to control the M-A351AS accelerometer for high-sensitivity measurement for the third design challenge. The M-A351 driver’s main functions can be broken down into three major tasks: power-on initialization, sensor measurement read, and switching to the IDLE mode (power-down sequencing).

The driver should follow the rules of register programming. Table 3 shows the registers which can control the accelerometer. The initial values of the control registers after internal initialization are the values indicated in the “Default” column. The registers can only be accessed when the device is in the mode specified in the “Mode” column. The host device must not attempt to access a register outside of its supported operation mode. Writing to a read-only register is prohibited, as shown in the column R/W.

The structure of driver code mainly depends on the method of data transmission. The selected version of the M-A351AS accelerometer uses the SPI bus for data transmission. SPI is a synchronous serial communication interface specification used primarily in embedded systems. SPI devices communicate in full duplex mode using a master-slave architecture with a single master. The master device originates the frame for reading and writing. Multiple slave devices are supported through selection with individual chip select (CS) lines. The SPI bus specifies five logic signals: (1) SCLK—Serial Clock (output from master); (2) MOSI—Master Output Slave Input, or Master Out Slave In (data output from master); (3) MISO—Master Input Slave Output, or Master In Slave Out (data output from slave); (4) SDIO—Serial Data I/O (bidirectional I/O), which is usually used to know whether data transmission has been ready or not, called DRDY; (5) CS—Chip Select (often active low, output from master).

**Figure 7:**
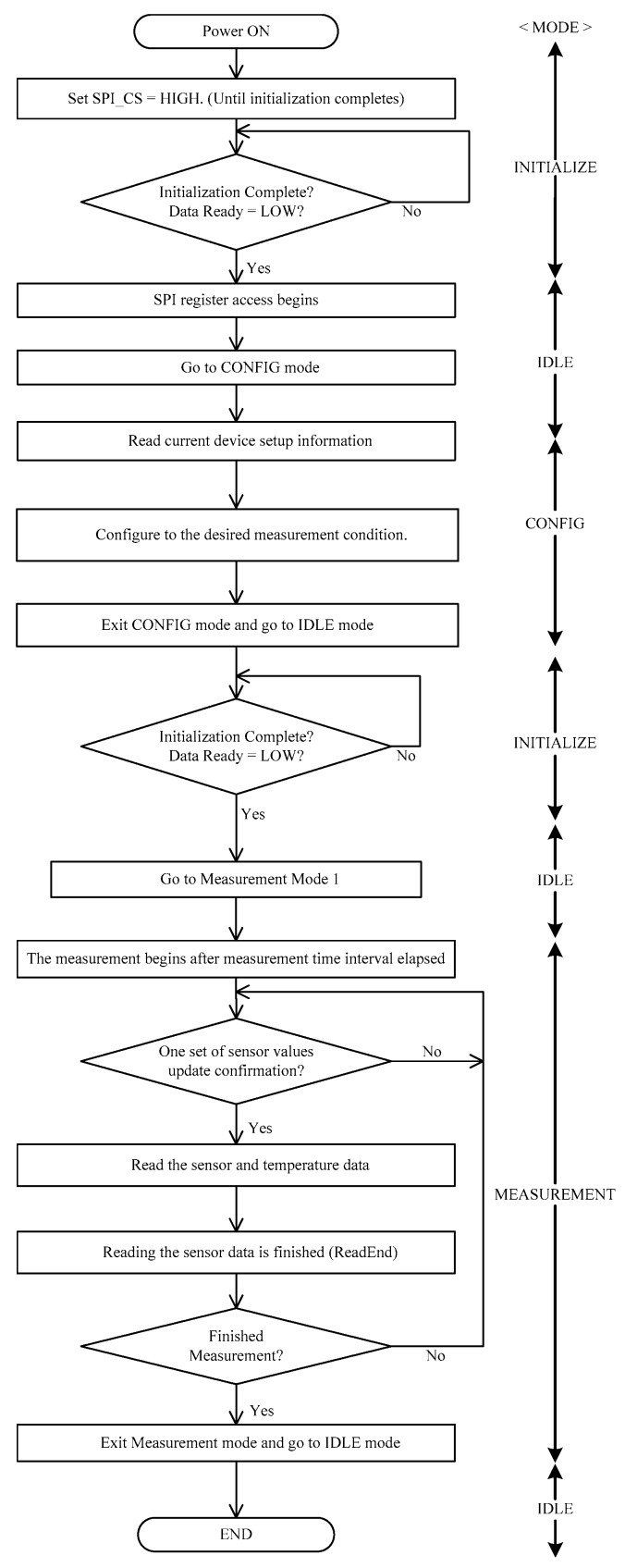
Flowchart of driver of M-A351AS [27].

Figure 7 shows the control flow of the driver code by the use of registers, including the three main tasks of M-A351AS: initialization, sensor measurement read, go to IDLE (power-down). 

According to the datasheet of M-A35AS [27], the CS pin should be kept high for at least 2 s (for internal initialization) when the accelerometer is being powered on through the switch. After the voltage of the DRDY pin changes from high to low, it indicates that the M-A351AS has been initialized correctly. Firstly, the M-A351AS enters the Config mode from the IDLE mode. Trigger mode, sampling rate, data type and filter parameters can be set in the Config mode. These initialization settings are application-specific and depend on requirements such as the expected frequency range of the sensing bandwidth, sensor data storage capacity of the host system, and power consumption. For example, the M-A351AS supports a selectable output rate from 50 to 500 samples/second and selectable filter cut-off frequency from 5 to 50 Hz. Typically, the goal of the driver software design is a trade-off to optimize to achieve the highest output rate, highest sensing bandwidth, lowest storage requirement, and lowest current consumption.

After configuration, the M-A351AS comes back to the IDLE mode again for an intermediary re-initialization. The M-A351AS then enters the Measurement mode for obtaining the sensor data. The changing level of the DRDY pin reflects the sampling rate set in the Config mode. Data acquisition is conducted when the DRDY pin is high. The data can include the tri-axial acceleration, tilt, or tilt speed, as well as the temperature. Following completion of the sensor data reading phase, the M-A351AS can be placed in the IDLE mode and be safely powered-down to reduce current draw.

Based on these hardware and software developments, the next section will focus on experimental validation of the developed high-sensitivity wireless accelerometer.

## 4. Experimental Validation

The performance of the new wireless accelerometer is evaluated experimentally through two types of tests: high-level excitation test and ambient vibration test. The high-level excitation tests were conducted on a shaking table in order to demonstrate the measurement capability of the sensor for high-amplitude vibration; ambient vibration tests were performed both in the laboratory and in the field to demonstrate sensor performance for low-frequency and low-amplitude vibration. This section provides a detailed description and discussion of these tests.

### 4.1. High-Level Excitation Test on Shaking Table

**Figure 8:**
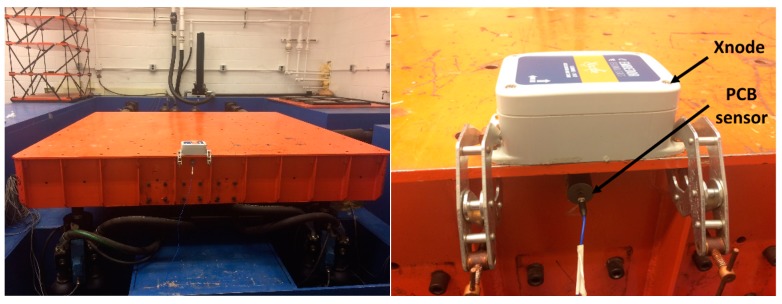
Shaking table test.

The high-level excitation test of wireless accelerometer was conducted at the Smart Structures Technology Laboratory, University of Illinois at Urbana-Champaign (UIUC). A 6-DOF shaking table was used to generate high-level excitation, which is controlled by a SC-6000 servo control system (Shore Western, Monrovia, CA, USA) as shown in Figure 8. 

The control system generated a band-limited white noise excitation; the maximum amplitude was around 0.5 g peak, with a bandwidth of 200 Hz. A wired piezoelectric accelerometer, model PCB393B04, was considered as a reference sensor. The PCB393B04 sensor has excellent performance for seismic monitoring, including a wide-range measurement of ±5 g, and low noise level of 3 μg/rms over 0.06~450 Hz. An m + p spectrum analyzer, VibPilot (m+p International, Verona, NJ, USA), which has 24-bit ADC, served as a data acquisition system for the wired sensor; a sampling frequency of 512 Hz was employed. The M-A351AS was configured with a 500 Hz sampling frequency; the cutoff frequency and the number of taps in the internal FIR filter were set to be 50 Hz and 512, respectively, which results in a 3 dB roll-off at 50 Hz.

**Figure 9:**
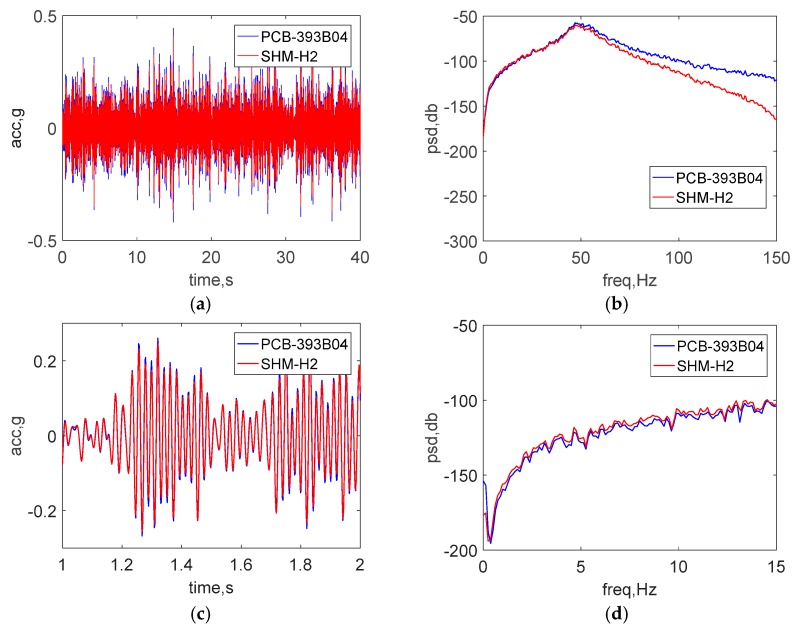
Shaking table test results: (**a**) time history data (**b**) PSD data (**c**) zoomed view of time history data (**d**) zoomed view of low-frequency PSD data.

Figure 9a,b show a comparison of the raw acceleration data in the both time and frequency domain. To make a direct comparison in the time domain, the data was sent through an 8-pole elliptic low-pass filter with a cutoff frequency of 50 Hz and is displayed in Figure 9c. Additionally, Figure 9d shows the power spectral density (PSD) in low frequency domain from 0 to 15 Hz. The excellent agreement between the results of two sensors demonstrate the ability of the wireless accelerometer to measure accurately high-amplitude accelerations.

### 4.2. Ambient Vibration Test in the Laboratory

**Figure 10:**
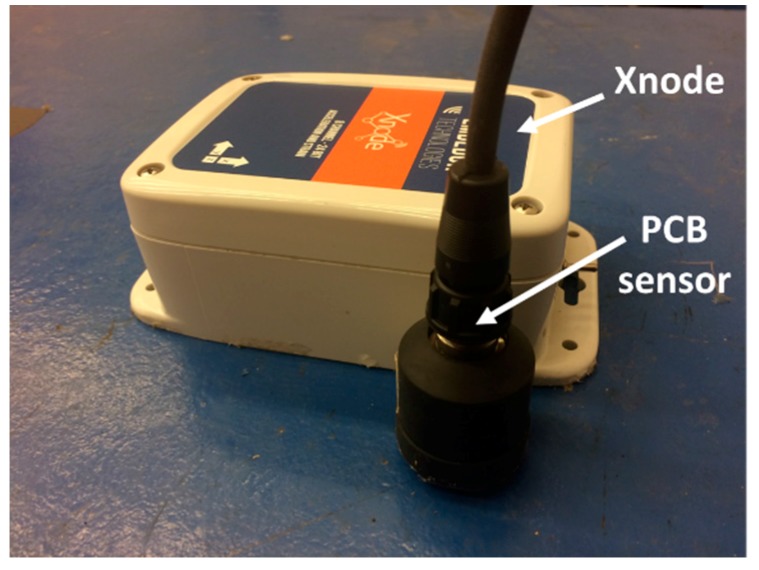
Ambient vibration test in basement.

The performance of the new wireless accelerometer was then evaluated under low-level ambient vibration conditions on a 41-ton concrete mass in the basement of the Newmark Civil Engineering Laboratory, UIUC, as shown in Figure 10. The ambient vibration test was conducted at night when students were not present and most machines and air conditioners in the laboratory were turned off. A wired high-sensitivity piezoelectric accelerometer, model PCB393B12 (PCB Piezotronics, Inc., Depew, NY, USA), was considered as a reference sensor. The PCB393B12 sensor has excellent features to enable low-level vibration measurement, including a high-sensitivity of 10 V/g, and low noise level of 8 μg/rms over 0.15~1000 Hz. The sampling frequencies of the wired PCB sensor and the wireless accelerometer were 512 Hz and 500 Hz, respectively. A total of 100 s data was collected for subsequent analysis.

**Figure 11:**
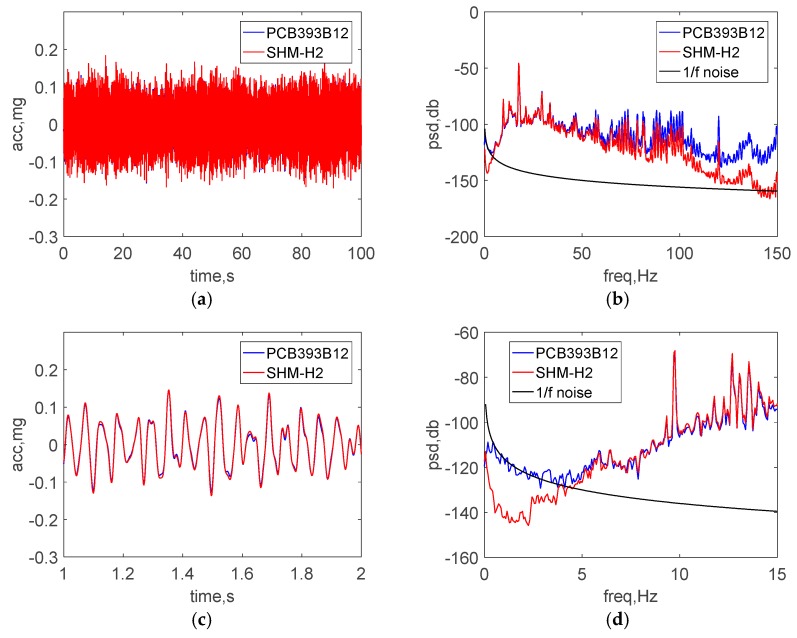
Test results in the basement: (**a**) time history data (**b**) PSD data (**c**) zoomed view of time history data (**d**) zoomed view of low-frequency PSD data.

Figure 11a,b show a comparison of the raw acceleration data in the both time and frequency domain under ambient vibration. As was done with the high-level excitation tests, the data shown in Figure 11c was passed through by an 8-pole elliptic low-pass filter with a cutoff frequency of 50 Hz to allow a direct comparison in the time domain. The peak amplitude of vibration is around 0.1 mg. Additionally, Figure 11d shows the PSD in low frequency domain from 0 to 15 Hz, where the 1/f noise in the PCB393B12 is seen. More specifically, 1/f noise is the dominant low-frequency noise for which the PSD is inversely proportional to the frequency. 1/f noise in accelerometers is often too small to be seen in typical measurement situations; however, it may have significant impact on low-frequency ultralow-level measurement. The PSD of the acceleration should approach zero (negative infinity on the dB-scale) with decreasing frequency. Therefore, the non-zero PSD obtained from the accelerometers in the low-frequency range is mainly due to 1/f noise in the wired accelerometer. The excellent agreement between the results of two sensors demonstrates the ability of the wireless accelerometer to measure accurately low-level accelerations. In particular, the new accelerometer has much smaller noise than the wired sensor, which can be seen in ultra-low frequency domain of 0~5 Hz.

### 4.3. Ambient Vibration Test on Country Road

**Figure 12:**
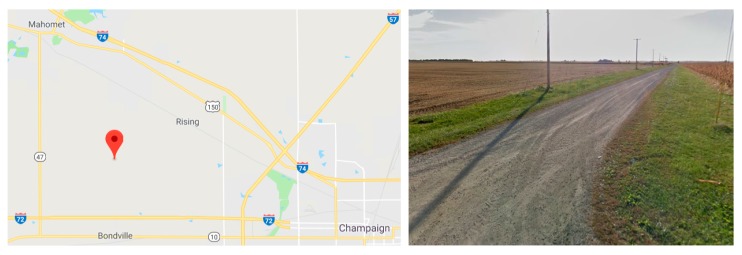
Ambient vibration test on country road.

The vibration level of the test in the laboratory in Section 4.2 and Section 4.3 is still relatively large, when compared to the capabilities of the new wireless accelerometer. Therefore, an additional ambient vibration test was conducted under ultralow-level vibration on a country road, between the cities of Mahomet and Champaign, Illinois (see Figure 12). The test site is chosen intentionally far away from main roads, to ensure the minimal effect of the vibration induced by vehicle traffic. The test was conducted early on a Sunday morning to further limit vehicle traffic. The wired PCB393B12 sensor was selected as the reference sensor. Both the wired PCB393B12 and the new wireless accelerometer were mounted on an 18 kg steel mass and placed on modeling clay on the country road. The test setup and sensing parameters were the same as those conducted in the laboratory.

**Figure 13:**
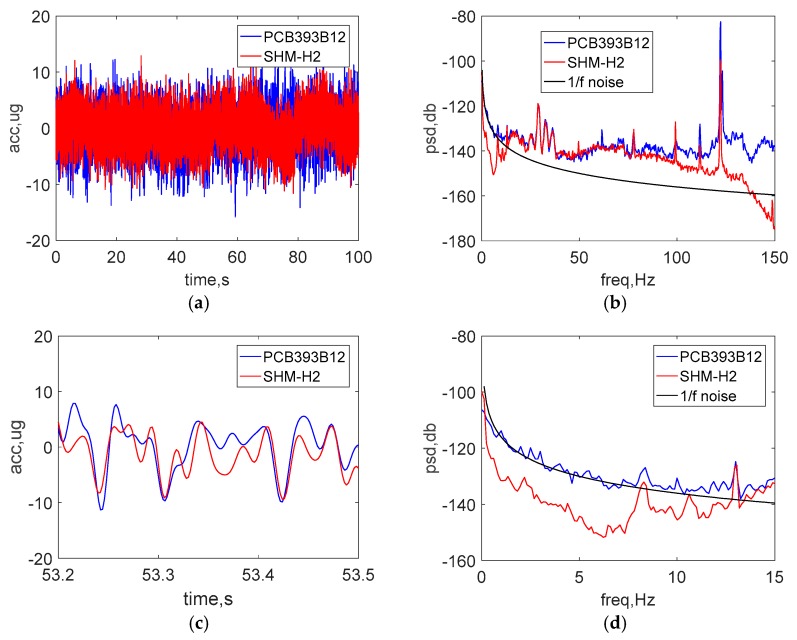
Test results on country road: (**a**) time history data (**b**) PSD data (**c**) zoomed view of time history data (**d**) zoomed view of low-frequency PSD data.

Figure 13a,b show a comparison of the raw acceleration data in the both time and frequency domain under ultralow-level ambient vibration. Good agreement can be observed between the wired sensor and the new wireless accelerometer at frequencies above 15 Hz. As was done with the high-level excitation tests, the data shown in Figure 13c was passed through an 8-pole elliptic low-pass filter with a cutoff frequency of 50 Hz to allow a direct comparison in the time domain. The peak amplitude of vibration is around 10 μg. 

Additionally, Figure 13d shows the PSD in low-frequency domain from 0 to 15 Hz, where the 1/f noise in the PCB393B12 is seen. In this test, the PSD in the entire low-frequency domain is smaller than the noise of PCB sensor; the evident difference of PSD between the two sensors demonstrates that the new wireless accelerometer has excellent capabilities, even for extremely low-amplitude vibration.

### 4.4. Ambient Vibration Test on Optical Isolation Table

To better assess the performance of the new wireless sensor, a more sensitive reference accelerometer was acquired. The accelerometer chosen was the PCB393B31, which has excellent low-level vibration measurement performance, with a sensitivity of 10 V/g and ultra-low noise level of 1 μg/rms over 0.1~200 Hz. The sampling frequencies of the wired PCB sensor and the wireless accelerometer were 512 Hz and 500 Hz, respectively. A total of 100 s data was collected for subsequent analysis.

**Figure 14:**
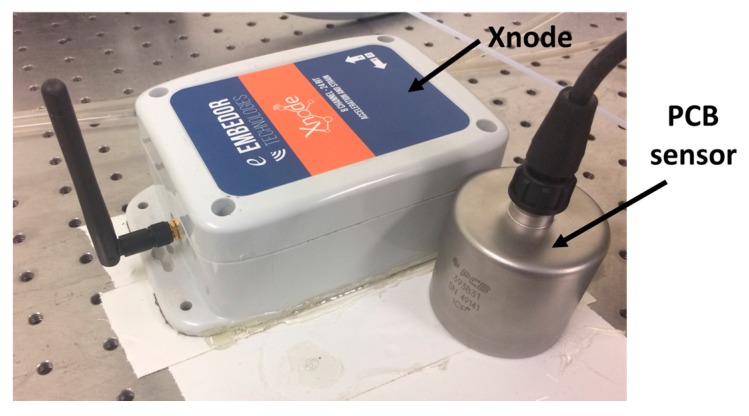
Ambient vibration test in an optical table.

Extreme cold weather in Illinois prevented testing outside; instead, testing was conducted on an optical isolation table in the Newmark Civil Engineering Laboratory, UIUC, as shown in Figure 14. This optical table incorporates vibration isolation and damping features to reduce the ambient vibration. Similar to the test in Section 4.2, the ambient vibration test was conducted at night to minimize the sources of ambient vibration.

**Figure 15:**
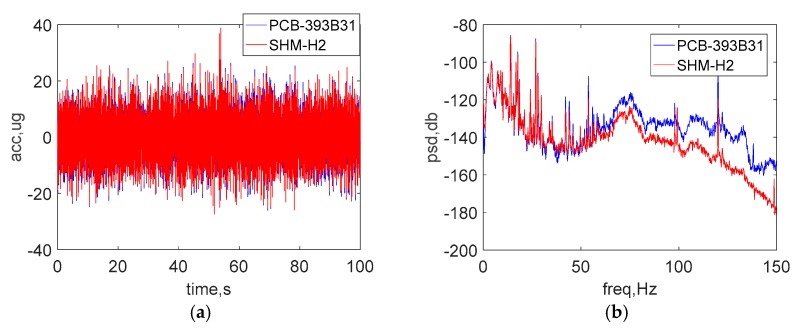

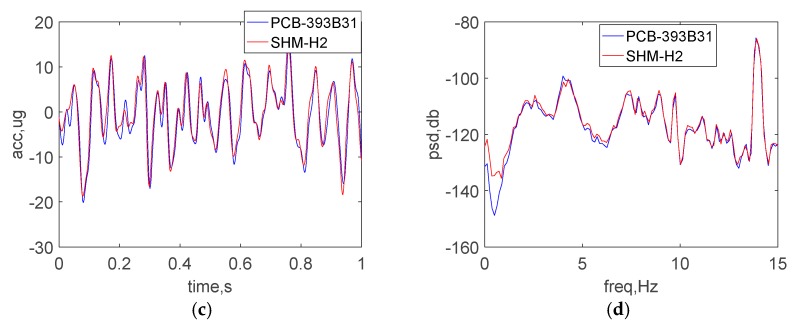
Test results on an optical table: (**a**) time history data (**b**) PSD data (**c**) zoomed view of time history data (**d**) zoomed view of low-frequency PSD data.

Figure 15a,b show a comparison of the raw acceleration data in the both time and frequency domain under ambient vibration. As was done with the previous tests, the data shown in Figure 15c was passed through an 8-pole elliptic low-pass filter with a cutoff frequency of 50 Hz to allow a direct comparison in the time domain. The peak amplitude of vibration is around 20 μg. Additionally, Figure 15d shows the PSD in low-frequency domain from 0 to 15 Hz. The excellent agreement between the results of two sensors demonstrates the ability of the wireless accelerometer to measure accurately low-level accelerations. In particular, the noise level of the new accelerometer is close to that found in the wired sensor, which can be seen in ultra-low frequency domain of 0~2 Hz.

The results in this section demonstrate the ability of the developed wireless accelerometer to measure accurately high-amplitude accelerations, as well as extremely low-amplitude accelerations, and meet the requirements for effective monitoring of civil infrastructure.

## 5. Conclusions

This paper details the development and validation of a high-sensitivity wireless accelerometer based on the Xnode platform. Diverse considerations for the low-noise and high-sensitivity implementation were addressed, and the performance was tested and validated through large-amplitude forced vibration and low-amplitude ambient vibration tests. Excellent agreement was found between the new wireless accelerometer and the high-sensitivity wired piezoelectric accelerometers, demonstrating the efficacy of the developed node. This new high-sensitivity wireless accelerometer will extend the use of WSSN to a broader class of structural health monitoring applications.

## Figures and Tables

**Table 1:** Summary of MEMS-based accelerometers in WSS nodes.

Accelerometer Model	Interface	Noise-Density (μg/$\sqrt{Hz}$)	Voltage Source (V)	Sensitivity (V/g)	Range (g)	BW (Hz)	Study
SD-1221	Analog	5	5	2.0	±2	0~400	[7,12,13,14,15]
CXL01LF	Analog	70	6~30	2.0	±1	0~50	[16]
CXL02LF	Analog	140	6~30	1.0	±2	0~50	[17]
AC310-002	Analog	10	9~20	2.0	±2	0~300	[18]
LIS2L02AL	Analog	30	3.3	0.66	±2	0~100	[19]
LIS2L06AL	Analog	30	3.3	0.66/0.22	±6/±2	0~100	[20]
LIS3L02AS4	Analog	50	3.3	0.66	±2	0~50	[21]
LIS344ALH	Analog	50	3.3	0.66/0.22	±6/±2	0~1800	[22]
LIS3L02DQ	Digital	110	3.3	1024LSB/g	±2	0~1120	[23,24]
SF1500	Analog	0.3	±6~±15	1.2	±3	0~1500	[8,9]
SF1600	Analog	0.3	±6~±15	1.2	±3	0~1500	[10,11]

**Table 2:** M-A351 accelerometer specification [27].

Parameter	Value
Acceleration range	±5 g
Temperature range	−20 to 85 °C
Width bandwidth	100 Hz
Resolution	0.06 μg/LSB
Output noise	0.5 μg/$\sqrt{\mathrm{Hz}}$
Operating voltage	3.3 V
Power consumption	66 mW (20 mA)
Output mode	Acceleration, Tilt angle, Tilt angle speed
Dynamic range	123 dB (approx.)
Digital serial interface	SPI and UART
Maximum sampling rate	500 sps (SPI) or 1000 sps (UART)

**Table 3:** Register map of the M-A351AS accelerometer [27].

Name	R/W	Mode	Address	Default	Function
XACCD_OUT	R	Measurement	0x00-0x03		Acceleration Sensor X Axis Value
YACCD_OUT	R	Measurement	0x04-0x07		Acceleration Sensor Y Axis Value
ZACCD_OUT	R	Measurement	0x08-0x0B		Acceleration Sensor Z Axis Value
TRIGR_CTRL	R/W	Config	0x20	0x20	Data Output Trigger Control
INTVL_TIME	R/W	Config	0x21	0x05	Timer Interval Value
MSC_CTRL	R/W	Config	0x23	0x00	Sensor type Control
PYCL_CTRL	R/W	Config	0x24	0x05	Physical Output Control
MODE_CTRL	R/W	ALL	0x25	0x00	Operation Mode Control
FILTER_CTRL	R/W	Config	0x28	0x65	Internal Filter Control
TEMP_OUT	R	Measurement	0x29-0x2B		Internal Temperature Sensor Value
MODEL_READ	R	Config	0x30-0x50		Read Product Information
SLFTEST_READ	R	Config	0x51		Read Self-diagnosis Results
BIAS_CTRL	R/W	Config	0x52-0x57	0x00	Bias Correction Control
RESET_CTRL	R/W	ALL	0x60	0x00	Software Reset
